# ONLINE MINDFULNESS PROGRAM FOR LONELY OLDER ADULTS

**DOI:** 10.1093/geroni/igz038.660

**Published:** 2019-11-08

**Authors:** Rachel Ungar, Lizi Wu, Karen Keown, James Schaeffer, Ellen Wicker

**Affiliations:** 1 Optum, Ann Arbor, Michigan, United States; 2 UnitedHealthcare, Minneapolis, Minnesota, United States; 3 AARP Services, Inc., Washington, District of Columbia, United States

## Abstract

Mindfulness meditation is a cognitive state of self-awareness that promotes emotional regulation and change in self-perspective. Mindfulness has been applied to address loneliness, stress, and anxiety, demonstrating consistent health benefits. The purpose of this study was to test the feasibility of an online mindfulness program and to measure its impact on well-being among lonely older adults. The intervention consisted of seven one-hour weekly online modules led by a trained facilitator via WebEx. Engagement was high with 63% of participants attending four or more sessions. Pre/post survey data (N=42) found decreased anxiety, stress, and improvement in mindfulness, purpose in life, and resilience. This program demonstrates that online mindfulness programs may be of great benefit for lonely older adults. Future research will include larger samples to investigate further impacts.

